# Identifying All Internal Forces in Existing Reinforced Concrete Components Using the Stress Release Method

**DOI:** 10.3390/ma18061300

**Published:** 2025-03-15

**Authors:** Liang Lu, Minghao Yin, Wanqiu Xia, Musaab Suliman, Lei Wang

**Affiliations:** 1 State Key Laboratory of Disaster Reduction in Civil Engineering, Tongji University, Shanghai 200092, China; 95010@tongji.edu.cn (L.L.); yinminghaozsa@163.com (M.Y.); musaab@tongji.edu.cn (M.S.); 2010027@tongji.edu.cn (L.W.); 2 Department of Disaster Mitigation for Structures, Tongji University, Shanghai 200092, China

**Keywords:** stress release method, existing reinforced concrete component, internal force identification, loading test, grooving method

## Abstract

The internal force state in concrete components is a crucial factor in evaluating the safety performance of existing buildings, bridges, and other concrete structures, while theoretical and numerical analysis of an ideal model may not accurately capture the actual internal forces within concrete components. This study introduces the basic principles of stress release technology for identifying internal forces in existing reinforced concrete components and provides a detailed derivation of normal and shear strains of component sections under each internal force component. It demonstrates that the internal forces of reinforced concrete sections can be accurately identified by testing the strain on the midpoint of three surface sides. A finite element model is established to investigate the relationship between groove depth and groove side length when normal or shear stress is released to zero, as well as the impact of reinforcement ratio on the stress release level. Experimental research is conducted using the grooving method to identify internal forces in reinforced concrete components under different external loads. The test results exhibit strong agreement with numerical simulation results. Additionally, the identification errors for axial forces and bending moments are within 10%, underscoring the feasibility of measuring internal forces in existing reinforced concrete components through the stress release method.

## 1. Introduction

The total area of existing buildings in China exceeds 20 billion square meters, with over 15% requiring detection, identification, and strengthening due to various factors, such as design, construction, and life cycle considerations [1,2]. In developed western countries, construction maintenance and strengthening costs account for approximately 50% of total investment in civil construction. Accurately evaluating the internal forces within concrete structures is crucial for ensuring the safety and reliability of buildings, bridges, and other concrete infrastructures [3,4,5]. Throughout the lifespan of concrete structures, factors such as stress attenuation, concrete creep, construction deviation, and uneven settlement lead to variations in forces, often making theoretical and numerical calculations of internal forces within a structure diverge from actual forces. Consequently, structural strengthening based solely on calculation results may not guarantee the seismic bearing capacity of the structure [6]. Therefore, the development of an accurate method for measuring internal forces in structural components is of critical importance.

Machine learning (ML) has emerged as a powerful approach for predicting the mechanical properties of concrete structures, surpassing traditional empirical methods by capturing complex nonlinear relationships [7]. Among ML models, extreme gradient boosting (XGB) and deep neural networks (DNNs) have demonstrated notable predictive performance. XGB is well suited for small to medium datasets, offering high accuracy and efficient parallel processing, though it requires careful hyperparameter tuning and is sensitive to data quality [8]. DNNs excel in modeling complex relationships and achieving superior predictive accuracy, but their reliance on large datasets and substantial computational resources limits their applicability in industrial settings [9]. Challenges remain in ensuring data quality, optimizing model parameters, and scaling these methods for real-world applications [10,11].

The stress release method is effective for measuring internal stress in components, which has gained widespread concern in academia and industry during the past few decades [12,13,14,15,16,17,18,19]. This method encompasses various techniques, such as the hole-drilling, core-drilling, and grooving methods. The hole-drilling method, initially introduced by Mathar in 1934 [20], has been widely utilized for measuring residual stress in metal components [21,22,23]. Subsequent advancements by researchers [24,25,26] have refined this method into a comprehensive system theory, promoting the establishment of standardized testing procedures such as ASTM E837-20 [27] and GB/T 31310-2014 [28]. In recent years, there has been a growing interest in applying the hole-drilling method to concrete materials. Chang et al. [29] proposed a novel approach combining a digital discrete image processing photo-elastic coating and the hole-drilling method to determine axial residual stresses in pre-stressed concrete specimens. Alternatively, the heterogeneous nature of concrete materials often results in discrete test results, posing challenges for its application in existing structures [12].

Compare with the hole-drilling method, the core-drilling method which adopts a ring hole is more employed on concrete materials. Trautner et al. [30,31] introduced analytical formulations of the influence function (IF) specific to the core-drilling method in concrete structures and validated the technique’s accuracy through finite element simulations. Their findings demonstrated that the maximum error between calculated and applied stresses was less than 3%. Ruan et al. [32] optimized the combination of the core-drilling method and IF to determine uniaxial in site stresses within existing bridge concrete components. Their numerical and experimental analysis indicated that the stress identification accuracy was within 10%, confirming the feasibility of this approach. To enhance identification stability and accuracy, McGinnis et al. [33] integrated the core-drilling method with digital image correlation and considered factors, such as water, concrete shrinkage and steel reinforcement, revealing that the relative error of in situ stresses in experiments decreased from 10% to 28%. Deng et al. [34] investigated the effects of the borehole diameter, drilling depth, strain sensor size and borehole position to improve the accuracy of the core-drilling method in measuring in situ stresses within concrete beams.

The grooving method has emerged as an alternative to the core-drilling method for assessing working stresses in existing concrete structures, effectively addressing challenges associated with complicated wire connections and extensive damage to the concrete. Researchers like Daniel et al. [35] applied the square grooving method to measure stresses in airport roads. Their test results showed that the working stress of the road surface is fully released when the ratio of grooving depth to spacing is 0.53. Wang et al. [36] investigated the relationship between released stress and grooving depth by numerical simulation, and established relations of the groove induced disturbance strain with aggregate diameter, distance of the grooves and working stress. Xu et al. [6] put forward a step-by-step grooving method for measuring working stresses in concrete compressed components under unidirectional stress, in which the effects of groove shape, spacing, length and depth on the stress-release degree were considered by numerical simulation. Notably, current research primarily focuses on stress identification in concrete components under simple external loads, especially uniaxial compression, which cannot accurately reflect the complex internal force state of concrete in real-world conditions. Furthermore, the lack of theoretical guidance on stress release methods for testing working concrete components hinders the direct application of research findings in engineering practice. Therefore, in this paper all internal forces in existing reinforced concrete components were identified by the stress release method with comprehensive theoretical derivations and experimental validation.

The study introduces a stress release method as a novel approach for measuring all internal forces in existing reinforced concrete components. The theory of internal force identification through stress release technology is systematically developed, in which normal and shear strains of component sections under each internal force component are derived in detail. Subsequently, finite element simulations are performed to investigate the relationship between groove depth and groove side length when normal or shear stress is released to zero, and to evaluate the influence of reinforcement ratio on the stress release level. Finally, test results are presented for reinforced concrete components subjected to various external forces, validating the feasibility and accuracy of the stress release technology in practice.

## 2. Stress Release Method

**Figure 1:**
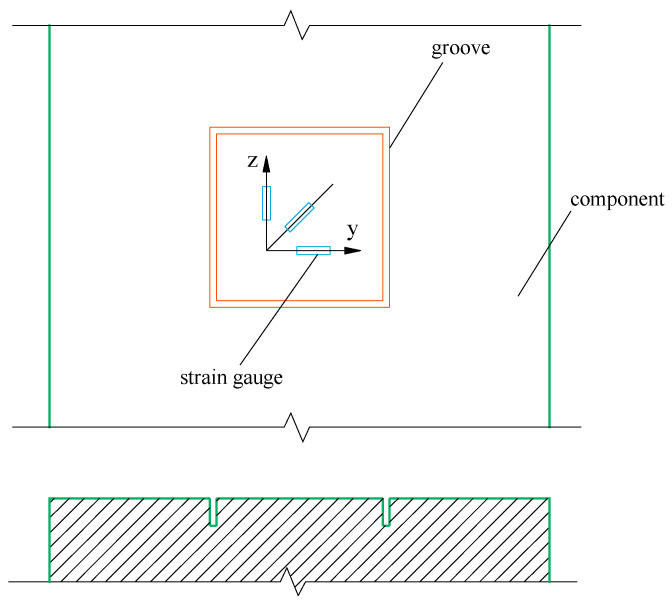
Pasting of strain gauges in the grooving method.

The stress release method involves creating one or several small holes or grooves on the surface of a component to release stress at the edge of the hole or groove. The released strain can be measured using electrical strain measurement techniques, allowing for the inverse estimation of the original stress at the measuring point. Various stress release methods, such as the hole-drilling, core-drilling, and grooving methods, can be employed for this purpose. The grooving method is increasingly utilized in practice due to its simpler wire connections and relatively minimal damage to the concrete. For instance, in the grooving method, four long grooves relieve the constraint at the measuring point where stress is released, causing the original stress field to become unbalanced. As a result, a certain amount of released strain, which is proportional to the released stress, is generated around the measuring point. The original stress field then reaches a new balance, forming new stress and strain fields. By measuring the released strain, the stress at the initial test point can be calculated using corresponding equations. The released strain measured by the strain gauges shown in Figure 1 can be expressed as follows:

(1) $$\varepsilon_{y}=\varepsilon_{0{}^{\circ}}$$

(2) $$\varepsilon_{z}=\varepsilon_{90{}^{\circ}}$$

(3) $$\gamma_{yz}=\varepsilon_{0{}^{\circ}}+\varepsilon_{90{}^{\circ}}-2\varepsilon_{45{}^{\circ}}$$

Once the strains of the measuring point are obtained, the stresses of this point can be calculated by using Hooke’s law in the plane stress state:

(4) $$\sigma_{y}=\frac{E}{1-\upsilon^{2}}(\varepsilon_{0{}^{\circ}}+\upsilon \varepsilon_{90{}^{\circ}})$$

(5) $$\sigma_{z}=\frac{E}{1-\upsilon^{2}}(\varepsilon_{90{}^{\circ}}+\upsilon \varepsilon_{0{}^{\circ}})$$

(6) $$\tau_{yz}=G(\varepsilon_{0{}^{\circ}}+\varepsilon_{90{}^{\circ}}-2\varepsilon_{45{}^{\circ}})$$

where *E*, ν and *G* are the elastic modulus, Poisson’s ratio and shear modulus of the tested material, respectively. It is observed that the results of the working stress test are mainly determined by two kinds of parameters, material properties and measured released strains.

## 3. Identifying Internal Forces in Existing Reinforced Concrete Components

**Figure 2:**
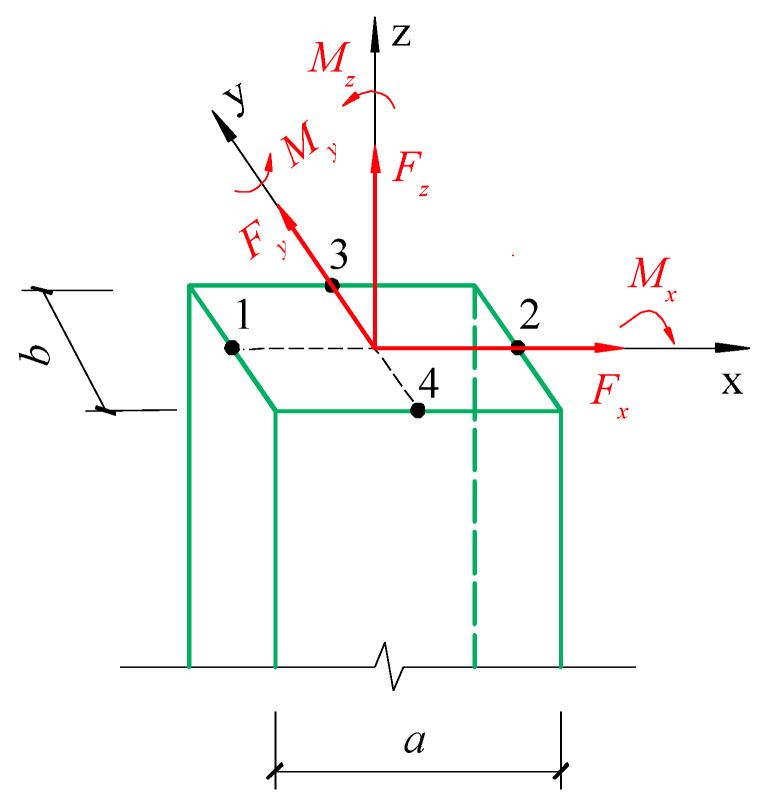
Force diagram of a rectangular section component.

Beam, column, plate, and wall are fundamental structural concrete components. Walls primarily experience in-plane shear force and bending moment, plates typically undergo in-plane axial force and out-of-plane bending moment, while beams and columns can have up to six internal force components, including axial force, double transverse shear force, double transverse bending moment, and torsion. In this section, as shown in Figure 2, a reinforced concrete column section is used as a case study to derive the normal and shear strains at the midpoint of the component surface under the action of internal forces. In the reversed way, the internal forces of the column section can be deduced by the released strains obtained through the testing, so as to prove the feasibility of the stress release technology.

### 3.1. Normal Stresses of Rectangular Section Under Axial Force and Bending Moment

Under the internal force *F_z_*, *M_x_* and *M_y_* as presented in Figure 2, only normal stresses are generated at the midpoints 1, 2, 3 and 4 of the section’s four sides:

(7) $$\left.\begin{array}{l} \sigma_{z,1}\\ \sigma_{z,2} \end{array}\right\}=\frac{F_{z}}{A}\pm \frac{M_{y}}{I_{y}}\times \frac{a}{2}$$

(8) $$\left.\begin{array}{l} \sigma_{z,3}\\ \sigma_{z,4} \end{array}\right\}=\frac{F_{z}}{A}\pm \frac{M_{x}}{I_{x}}\times \frac{b}{2}$$

where *a* and *b* are the side length of the section, *A* is the equivalent area of the section, *I_x_* and *I_y_* are the equivalent moment of inertia of the section. *A*, *I_x_* and *I_y_* can be calculated as follows:

(9) $$A=A_{c}+\frac{E_{s}-E_{c}}{E_{c}}A_{s}$$

(10) $$I_{x}=I_{c,x}+\frac{E_{s}-E_{c}}{E_{c}}\sum_{i=1}^{n}A_{i}{d_{i,y}}^{2}$$

(11) $$I_{y}=I_{c,y}+\frac{E_{s}-E_{c}}{E_{c}}\sum_{i=1}^{n}A_{i}{d_{i,x}}^{2}$$

where *A_c_* and *A_s_* are the area of concrete and longitudinal steel bars, *E_c_* and *E_s_* are the elastic modulus of concrete and steel bar, *I_c,x_* and *I_c,y_* are the concrete moment of inertia of the section, *n* is the number of steel bars, *A_i_* is the area of the *i*-th steel bar, and *d_i,x_* and *d_i,y_* are the distance from the centroid of the *i*-th steel bar to the cross-sectional neutral axis.

### 3.2. Shear Stress Distribution of Rectangular Section Without Reinforcement Under Torsion

The derivation of the shear stress in the reinforced section under torsion is relatively complicated. Firstly, the shear stress in the homogeneous material is addressed, followed by developing a modified method to obtain the shear stress in the reinforced section. Due to the warping of noncircular sections when twisted, the traditional plane section assumption no longer holds. Therefore, the torsion problem of noncircular section components must be addressed using a two-dimensional elastic mechanics approach.

**Figure 3:**
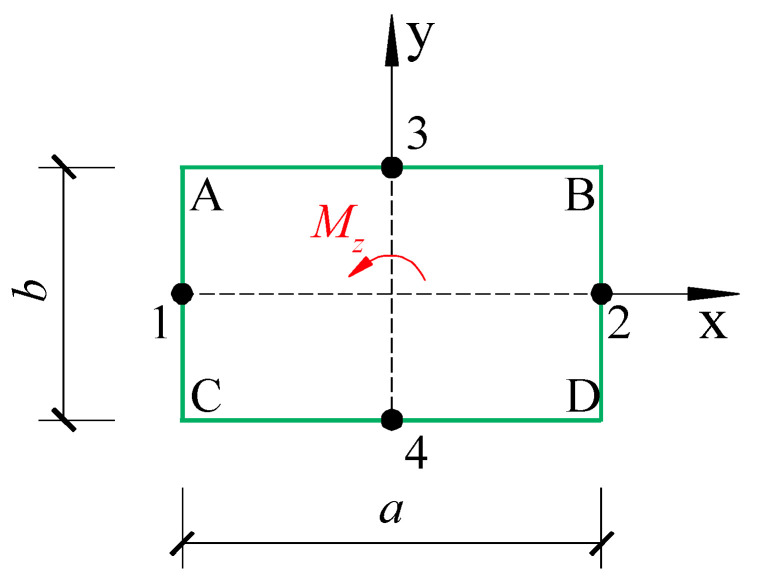
Rectangular section subjected to torsion.

Figure 3 shows the rectangular section subjected to torque *M_z_*, in which the shear stress can be expressed as [37]:

(12) $$\tau_{zx}(x,y)=-GK\left[2y-\frac{8b}{\pi^{2}}\sum_{m=1,3,5\ldots }^{\infty }\frac{{\left(-1\right)}^{\frac{m-1}{2}}\cosh\frac{m\pi x}{b}\sin\frac{m\pi y}{b}}{m^{2}\cosh\frac{m\pi a}{2b}}\right]$$

(13) $$\tau_{zy}(x,y)=GK\frac{8}{\pi^{2}}\sum_{m=1,3,5\ldots }^{\infty }\frac{{\left(-1\right)}^{\frac{m-1}{2}}\sinh\frac{m\pi x}{b}\cos\frac{m\pi y}{b}}{m^{2}\cosh\frac{m\pi a}{2b}}$$

where *G* is the shear modulus of the material,

(14) $$K=\frac{M_{z}}{Gab^{3}\left[\frac{1}{3}-\frac{64}{\pi^{5}}\frac{b}{a}\sum_{m=1,3,5\ldots }^{\infty }\frac{\tanh\frac{m\pi a}{2b}}{m^{5}}\right]}$$

Substituting y=±b/2 into Equation (12), the shear stress of AB and CD edges in Figure 3 can be obtained:

(15) $$\tau_{zx}(x,y=\pm \frac{b}{2})=\mp GKb\left[1-\frac{8}{\pi^{2}}\sum_{m=1,3,5\ldots }^{\infty }\frac{\cosh\frac{m\pi x}{b}}{m^{2}\cosh\frac{m\pi a}{2b}}\right]$$

Similarly, letting x=±a/2 in Equation (13), the shear stress of BC and AD edges can be calculated:

(16) $$\tau_{zy}(x=\pm \frac{a}{2},y)=\pm GK\frac{8b}{\pi^{2}}\sum_{m=1,3,5\ldots }^{\infty }\frac{{\left(-1\right)}^{\frac{m-1}{2}}\tanh\frac{m\pi a}{2b}\cos\frac{m\pi y}{b}}{m^{2}}$$

The maximum stress occurs at the midpoint of the long side. Letting x=0,y=±b/2 in Equation (12), the stresses at point 3 and point 4, which are the maximum stresses, are determined:

(17) $$\tau_{\max}=\tau_{zx}(x=0,y=\pm \frac{b}{2})=\mp GKb\left[1-\frac{8}{\pi^{2}}\sum_{m=1,3,5\ldots }^{\infty }\frac{1}{m^{2}\cosh\frac{m\pi a}{2b}}\right]$$

The maximum shear stress of the short side occurs at the midpoint of the short side. Substituting x=±a/2,y=0 into Equation (13), the shear stresses at point 1 and point 2 are calculated:

(18) $$\tau_{\max}^{\prime }=\tau_{zy}(x=\pm \frac{a}{2},y=0)=GK\frac{8b}{\pi^{2}}\sum_{m=1,3,5\ldots }^{\infty }\frac{{\left(-1\right)}^{\frac{m-1}{2}}\tanh\frac{m\pi a}{2b}}{m^{2}}$$

In practical applications, the following is assumed:

(19) $$\beta_{1}=\frac{\frac{1}{3}-\frac{64}{\pi^{5}}\frac{b}{a}\sum_{m=1,3,5\ldots }^{\infty }\frac{\tanh\frac{m\pi a}{2b}}{m^{5}}}{1-\frac{8}{\pi^{2}}\sum_{m=1,3,5\ldots }^{\infty }\frac{1}{m^{2}\cosh\frac{m\pi a}{2b}}}$$

(20) $$\upsilon_{1}=\frac{GK\frac{8b}{\pi^{2}}\sum_{m=1,3,5\ldots }^{\infty }\frac{{\left(-1\right)}^{\frac{m-1}{2}}\tanh\frac{m\pi a}{2b}}{m^{2}}}{GKb\left[1-\frac{8}{\pi^{2}}\sum_{m=1,3,5\ldots }^{\infty }\frac{1}{m^{2}\cosh\frac{m\pi a}{2b}}\right]}$$

Thus, Equations (17) and (18) are simplified as follows:

(21) $$\tau_{\max}=\frac{M_{z}}{\beta_{1}ab^{2}}$$

(22) $$\tau_{\max}^{\prime }=\upsilon_{1}\tau_{\max}$$

It is observed that the factors *β*_1_ and *υ*_1_ are only related to the ratio *a*/*b*, which can be calculated as shown in Table 1.

The analysis results from elastic mechanics indicate that the shear stress distribution on the cross section of a rectangular section component while subjected to torsion exhibits the following characteristics: (1) The shear stress direction at each point around the section is tangent to the periphery, and the shear stress at the corner of the section is zero; (2) The maximum shear stress occurs at the midpoint of the long side, while the shear stress at the midpoint of the short side is the maximum shear stress on the short side.

**Figure 4:**
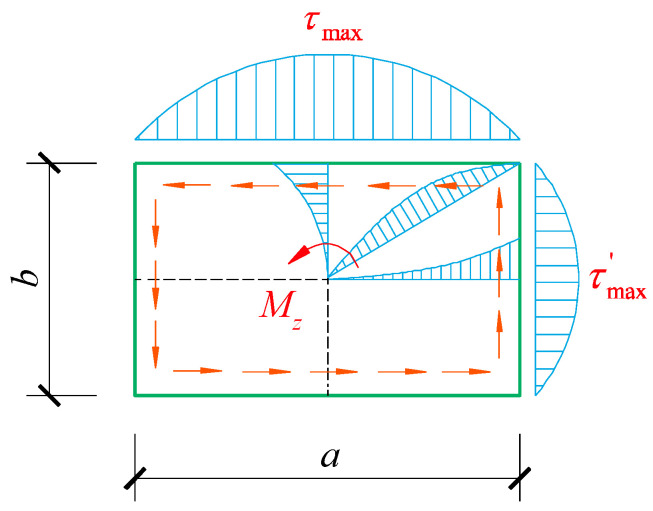
Shear stress distribution of rectangular section subjected to torsion.

The distribution of shear stress is shown in Figure 4 [38]. The maximum shear stress $\tau_{\max}$ along the long side and the maximum shear stress $\tau_{\max}^{\prime }$ along the short side can be calculated according to Equations (21) and (22).

### 3.3. Shear Stresses of Rectangular Section Without Reinforcement Under Shear Force

**Figure 5:**
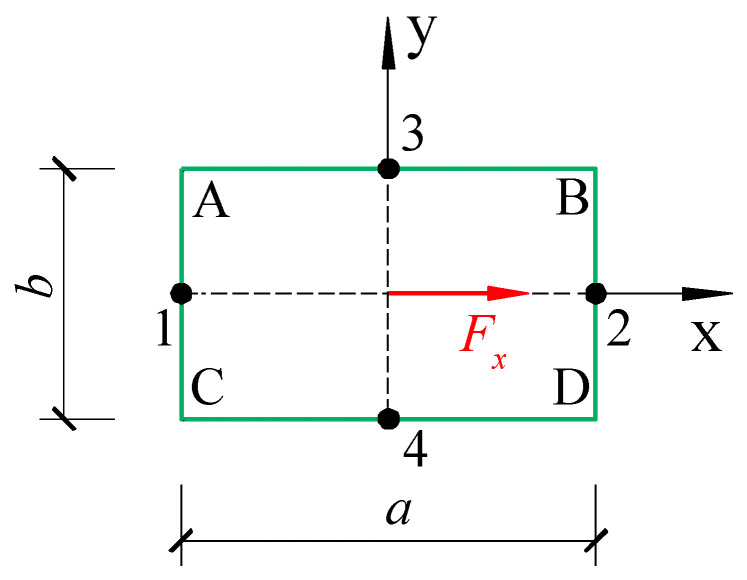
Rectangular section subjected to shear force.

Through the principles of elastic mechanics, shear stress expressions can be derived for the general rectangular section subjected to the shear force depicted in Figure 5 [39]:

(23) $$\tau_{zx}(x,y)=\frac{F_{x}}{2I_{y}}\left(\frac{a^{2}}{4}-x^{2}\right)+\frac{\upsilon }{1+\upsilon }\frac{F_{x}}{6I_{y}}\left(3y^{2}-\frac{b^{2}}{4}\right)-\frac{\upsilon }{1+\upsilon }\frac{F_{x}b^{2}}{2\pi^{2}I_{y}}\sum_{m=1}^{\infty }\frac{{\left(-1\right)}^{m}\cosh\frac{2m\pi x}{b}\cos\frac{2m\pi y}{b}}{m^{2}\cosh\frac{m\pi a}{b}}$$

(24) $$\tau_{zy}(x,y)=\frac{\upsilon }{1+\upsilon }\frac{F_{x}b^{2}}{2\pi^{2}I_{y}}\sum_{m=1}^{\infty }\frac{{\left(-1\right)}^{m}\sinh\frac{2m\pi x}{b}\sin\frac{2m\pi y}{b}}{m^{2}\cosh\frac{m\pi a}{b}}$$

The first part of Equation (23) represents the fundamental solution for the shear stress in the long and narrow rectangular section. It is evident from elastic mechanics calculations that when a/b≥2, the second and third parts of Equation (23) account for a small proportion, and the first part alone is sufficient for engineering accuracy. In contrast, when a/b≥2 is not satisfied, the contributions of the second and third parts of Equation (23) become significant and cannot be ignored.

Substituting x=0,y=±b/2 into Equation (23), the maximum shear stresses at point 3 and point 4 are obtained:

(25) $$\tau_{\max}=\tau_{zx}(x=0,y=\pm \frac{b}{2})=\frac{3F_{x}}{2ab}\left[1+\frac{\upsilon }{1+\upsilon }\frac{b^{2}}{a^{2}}\left(\frac{2}{3}-\frac{4}{\pi^{2}}\sum_{m=1}^{\infty }\frac{1}{m^{2}\cosh\frac{m\pi a}{b}}\right)\right]$$

Letting x=±a/2,y=0 in Equation (24), the shear stresses at point 1 and point 2 are calculated as follows:

(26) $$\tau_{zy}(x=\pm \frac{a}{2},y=0)=0$$

In practical application, the following is assumed:

(27) $$\lambda =\frac{4}{\pi^{2}}\sum_{m=1}^{\infty }\frac{1}{m^{2}\cosh\frac{m\pi a}{b}}$$

Therefore, Equation (25) can be simplified as follows:

(28) $$\tau_{\max}=\frac{3F_{x}}{2ab}\left[1+\frac{\upsilon }{1+\upsilon }\frac{b^{2}}{a^{2}}\left(\frac{2}{3}-\lambda \right)\right]$$

For the convenience of application, λ, corresponding to different aspect ratios *a*/*b*, is tabulated, as shown in Table 2.

When the external shear force *F* is parallel to the *y* axis, similar equations can be derived for the *x* axis. If the external force *F* passes through the bending center but is not parallel to the *x* or *y* axis, it can be decomposed into components *F_x_* and *F_y_* along the *x* and *y* axes. In cases where the external force *F* does not pass through the bending center, bending-torsion coupling deformation occurs. To address this, the force *F* can be translated to the bending center, treated as oblique bending to calculate the bending stress, and then superimposed with the torsional stress caused by the moment of *F* about the bending center to obtain the total stress distribution in the section.

### 3.4. Shear Stresses of Rectangular Section Under Torsion and Shear Force

In reinforced concrete rectangular section components subjected to torsion, the steel bars and concrete deformation are coordinated in the elastic stage. The torsional resistance of the entire section can be divided into two parts: the steel bar’s torsional resistance and the concrete’s torsional resistance. Based on the shear stress in Equation (21) for rectangular section under torsion, the torsional contribution of the steel bars in the elastic stage can be approximately considered as shown in Equation (29):

(29) $$M_{z}=\tau_{\max}\beta_{1}ab^{2}+\frac{G_{s}-G_{c}}{G_{c}}\sum_{i=1}^{n}\tau_{i}A_{i}d_{i}$$

where *G_s_* is the shear modulus of steel bar, *G_c_* is the shear modulus of concrete, *n* is the number of steel bars, $\tau_{i}$ is the shear stress at the centroid of the *i*-th steel bar when the torque is applied to the homogeneous material rectangular section (hereinafter referred to as the nominal torsional shear stress of the steel bar), *A_i_* is the area of the *i*-th steel bar, and *d_i_* is the distance from the centroid of the *i*-th steel bar to the centroid of the section.

Supposing the location $\left(x_{i},y_{i}\right)$ of the *i*-th steel bar, and the nominal torsional shear stresses in both directions of the steel bar are $\tau_{zx}(x_{i},y_{i})$ and $\tau_{zy}(x_{i},y_{i})$, then:

(30) $$d_{i}=\sqrt{x_{i}^{2}+y_{i}^{2}}$$

(31) $$\tau_{i}=\sqrt{\tau_{zx}^{2}(x_{i},y_{i})+\tau_{zy}^{2}(x_{i},y_{i})}$$

where $\tau_{zx}(x_{i},y_{i})$ and $\tau_{zy}(x_{i},y_{i})$ are calculated as Equations (12) and (13), respectively. Defining

(32) $$\eta_{i}=\frac{\tau_{i}}{\tau_{\max}}$$

when the length–width ratio of the section is determined, $\eta_{i}$ is solely related to the position of the steel bar, which is called the torsional position factor of the steel bar, and Equation (29) can be expressed as:

(33) $$\tau_{\max}=\frac{M_{z}}{\beta_{1}ab^{2}+\frac{G_{s}-G_{c}}{G_{c}}\sum_{i=1}^{n}\eta_{i}A_{i}d_{i}}$$

According to the shear stress Equation (28) for the rectangular section under shear force, the shear contribution of steel bars is considered in the same manner when the reinforced concrete rectangular section component is under shear force, as shown in the following equation:

(34) $$F_{x}=\frac{2ab}{3\left[1+\frac{\upsilon }{1+\upsilon }\frac{b^{2}}{a^{2}}\left(\frac{2}{3}-\lambda \right)\right]}\tau_{\max}+\frac{G_{s}-G_{c}}{G_{c}}\sum_{i=1}^{n}\tau_{i,zx}A_{i}$$

where $\tau_{i,zx}$ is the shear stress in the *x* direction of the centroid position of the *i*-th steel bar (hereinafter referred to as the nominal shear stress of the steel bar) when the shear force is applied to the homogeneous material component, and is calculated according to Equation (21). Defining

(35) $$\lambda_{i}=\frac{\tau_{i,zx}}{\tau_{\max}}$$

$\lambda_{i}$ is only related to the position of the steel bar, which is called the shear position factor of the steel bar, and Equation (34) can be expressed as follows:

(36) $$\tau_{\max}=\frac{F_{x}}{\frac{2ab}{3\left[1+\frac{\upsilon }{1+\upsilon }\frac{b^{2}}{a^{2}}\left(\frac{2}{3}-\lambda \right)\right]}+\frac{G_{s}-G_{c}}{G_{c}}\sum_{i=1}^{n}\lambda_{i}A_{i}}$$

**Figure 6:**
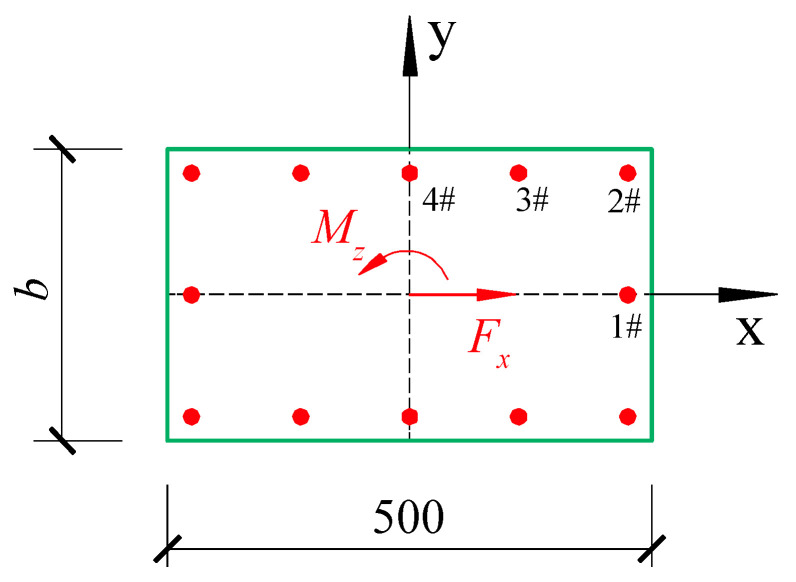
Steel bar position of rectangular section components.

The torsional position factor $\eta_{i}$ and the shear position factor $\lambda_{i}$ of the steel bar can be calculated using the equations provided above. For instance, consider a rectangular section with a length of *a* = 500 mm and a width of *b*, as illustrated in Figure 6. The steel bars are positioned as shown, with a diameter of 20 mm and a protective layer with a thickness of 30 mm. The calculation results of the torsional position factor and the shear position factor for the steel bars are presented in Table 3 and Table 4, respectively.

Under the assumption of small deformation, the shear stresses at points 1, 2, 3 and 4 in the section shown in Figure 2 can be obtained by the superposition principle as follows:

(37) $$\left.\begin{array}{l} \tau_{zy,2}\\ \tau_{zy,1} \end{array}\right\}=\upsilon_{1}\frac{M_{z}}{\beta_{1}ab^{2}+\frac{G_{s}-G_{c}}{G_{c}}\sum_{i=1}^{n}\eta_{i}A_{i}d_{i}}\pm \frac{F_{y}}{\frac{2ab}{3\left[1+\frac{\upsilon }{1+\upsilon }\frac{a^{2}}{b^{2}}\left(\frac{2}{3}-\lambda \right)\right]}+\frac{G_{s}-G_{c}}{G_{c}}\sum_{i=1}^{n}\lambda_{i}A_{i}}$$

(38) $$\left.\begin{array}{l} \tau_{zx,3}\\ \tau_{zx,4} \end{array}\right\}=-\frac{M_{z}}{\beta_{1}ab^{2}+\frac{G_{s}-G_{c}}{G_{c}}\sum_{i=1}^{n}\eta_{i}A_{i}d_{i}}\pm \frac{F_{x}}{\frac{2ab}{3\left[1+\frac{\upsilon }{1+\upsilon }\frac{b^{2}}{a^{2}}\left(\frac{2}{3}-\lambda \right)\right]}+\frac{G_{s}-G_{c}}{G_{c}}\sum_{i=1}^{n}\lambda_{i}A_{i}}$$

### 3.5. Internal Force Identification of Reinforced Concrete Rectangular Section Component

The internal force $F_{z}$, $M_{x}$ and $M_{y}$ can be determined by combining any three formulas of normal stress $\sigma_{z,1}$~$\sigma_{z,4}$ in Equations (7) and (8), taking $\sigma_{z,1}$, $\sigma_{z,2}$ and $\sigma_{z,3}$ as examples to calculate $F_{z}$, $M_{x}$ and $M_{y}$:

(39) $$F_{z}=\frac{1}{2}A\left(\sigma_{z,1}+\sigma_{z,2}\right)$$

(40) $$M_{x}=\frac{I_{x}}{b}\left(2\sigma_{z,3}-\sigma_{z,1}-\sigma_{z,2}\right)$$

(41) $$M_{y}=\frac{I_{y}}{a}\left(\sigma_{z,1}-\sigma_{z,2}\right)$$

Similarly, $M_{z}$, $F_{x}$ and $F_{y}$ can be obtained by combining any three formulas of shear stress $\tau_{zy,1}$, $\tau_{zy,2}$, $\tau_{zx,3}$ and $\tau_{zx,4}$ in Equations (37) and (38), taking $\tau_{zy,1}$, $\tau_{zy,2}$ and $\tau_{zx,3}$ as examples to calculate $M_{z}$, $F_{x}$ and $F_{y}$:

(42) $$M_{z}=\frac{\beta_{1}ab^{2}+\frac{G_{s}-G_{c}}{G_{c}}\sum_{i=1}^{n}\eta_{i}A_{i}d_{i}}{2\upsilon_{1}}\left(\tau_{zy,1}+\tau_{zy,2}\right)$$

(43) $$F_{x}=\left(\frac{2ab}{3\left[1+\frac{\upsilon }{1+\upsilon }\frac{b^{2}}{a^{2}}\left(\frac{2}{3}-\lambda \right)\right]}+\frac{G_{s}-G_{c}}{G_{c}}\sum_{i=1}^{n}\lambda_{i}A_{i}\right)\left(\tau_{zx,3}+\frac{\tau_{zy,1}+\tau_{zy,2}}{2\upsilon_{1}}\right)$$

(44) $$F_{y}=\left(\frac{2ab}{3\left[1+\frac{\upsilon }{1+\upsilon }\frac{a^{2}}{b^{2}}\left(\frac{2}{3}-\lambda \right)\right]}+\frac{G_{s}-G_{c}}{G_{c}}\sum_{i=1}^{n}\lambda_{i}A_{i}\right)\frac{\tau_{zy,2}-\tau_{zy,1}}{2}$$

The stress in the above equations can be calculated by the strain measured by the strain rosette through Equations (4)~(6); The coefficients *β_1_* and *υ_1_* can be obtained from Table 1, and the coefficients *λ*, *η_i_* and *λ_i_* can be obtained from Table 2, Table 3 and Table 4, respectively. Additionally, *a* and *b* represent the lengths of the long and short sides of the rectangular section, respectively, and $d_{i,x}$ and $d_{i,y}$ represent the distances from the *i*-th steel bar to the corresponding neutral axis.

It is evident from the above derivation that as long as the surface stresses at any three points of the 1~4 points on the section shown in Figure 2 are obtained using the stress release method, all the internal force components of the section can be calculated.

## 4. Test and Numerical Simulation

### 4.1. Test Specimens

**Figure 7:**
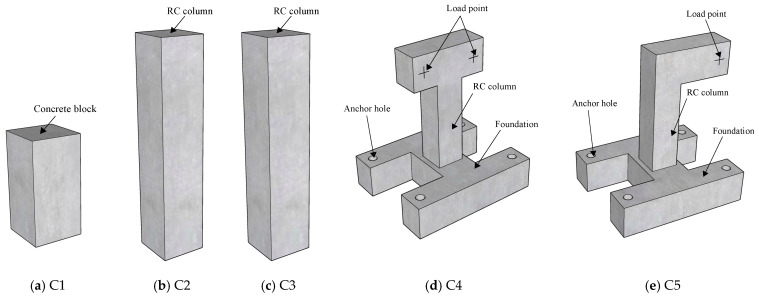
Configuration of test specimens.

**Figure 8:**
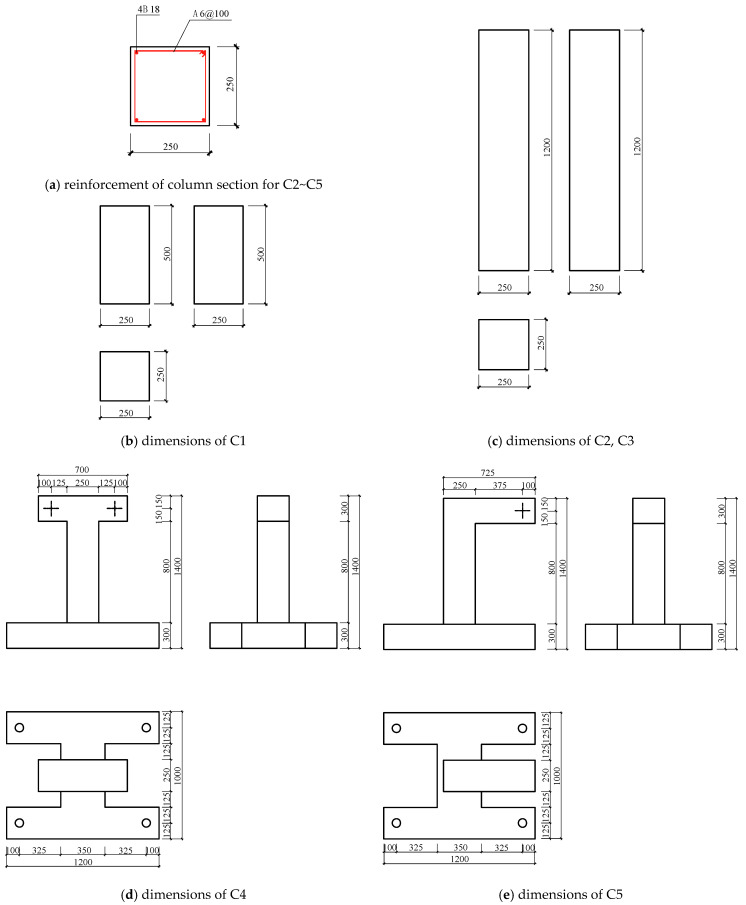
Design details of test specimens.

Based on the aforementioned formulas, the grooving method is employed to determine internal forces in concrete components, as it offers greater practicality and ease of implementation compared to the core-drilling method. A total of five groups (three specimens in each group) of reinforced concrete column specimens labeled as C1 to C5 were designed and tested. The configuration and design details of the specimens are shown in Figure 7 and Figure 8. The section size of the columns is 250 mm × 250 mm, and the height is 500 mm, 1200 mm and 1400 mm, respectively. The reinforcement detail of columns is shown in Figure 8a. The longitudinal bars are made of HRB335 steel with a yield strength of 335 MPa, while the stirrups are made of HPB300 steel with a yield strength of 300 MPa. The columns are constructed using C20, C30 and C40 concrete with a cubic compressive strength of 20 MPa, 30 MPa and 40 MPa, respectively, as presented in Table 5. The mechanical properties of the concrete and longitudinal bars were measured prior to testing and used in the numerical simulations. The measured values are presented in Table 6.

Table 5 also illustrates the force state, design load and test objectives for groups C1~5. These groups are used to (1) examine the correlation between the machine cutting disturbance and concrete strength, (2) analyze the relationship between the released strain and the geometric dimensions of the groove, and (3) compare the results of internal force identification with the applied load. Moreover, during the testing process, mechanical parameters, such as strength and elastic modulus of the specimens, are measured and recorded for further analysis.

### 4.2. Test Setup

**Figure 9:**
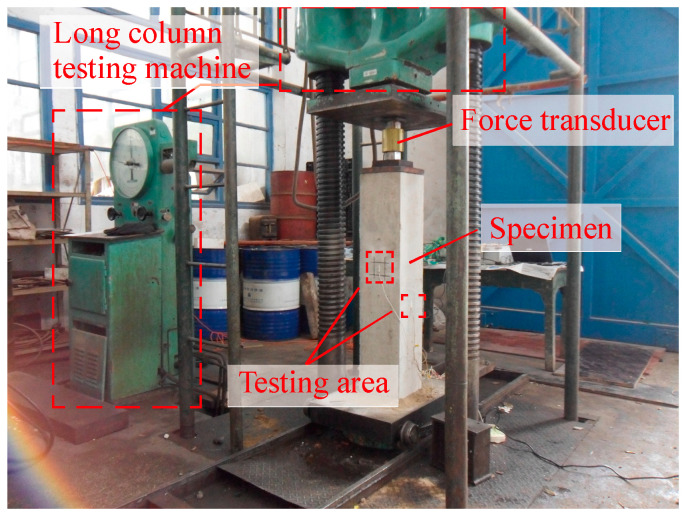
Axial compression test setup.

**Figure 10:**
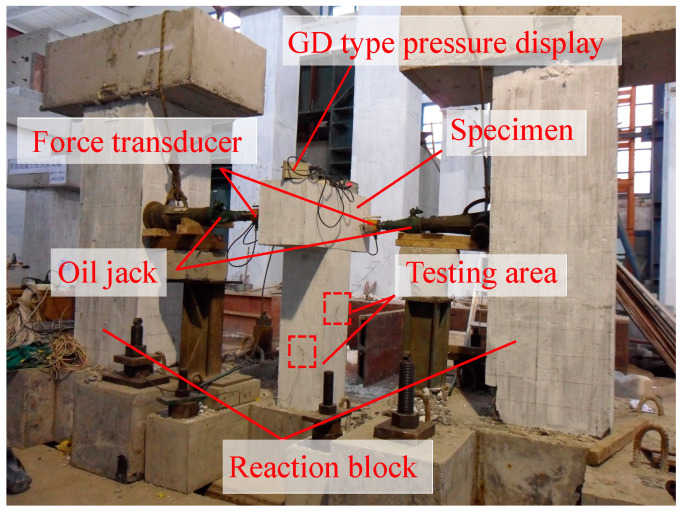
Pure torsion test setup.

**Figure 11:**
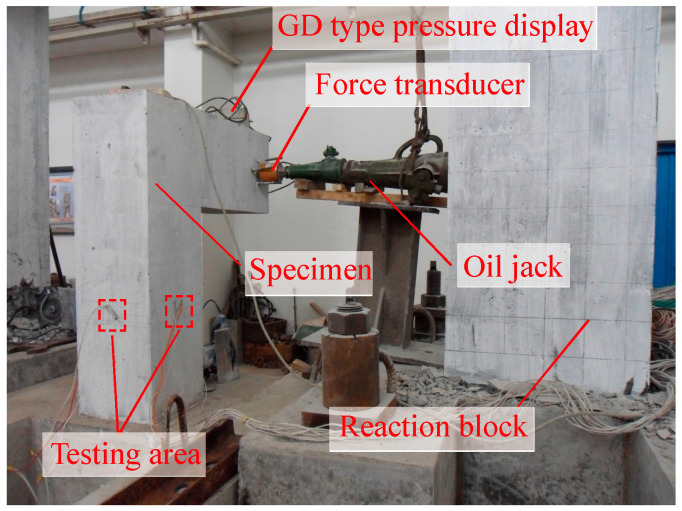
Bending-shear-torsion test setup.

Figure 9, Figure 10 and Figure 11 illustrate the test setups and specimens used in the study. The load was applied to specimens by a long column testing machine and several oil jacks. Force transducers and GD-type pressure displays were installed on specimens to measure and record the applied forces. A multi-channel static strain data acquisition system was utilized to collect data during the testing process.

**Figure 12:**
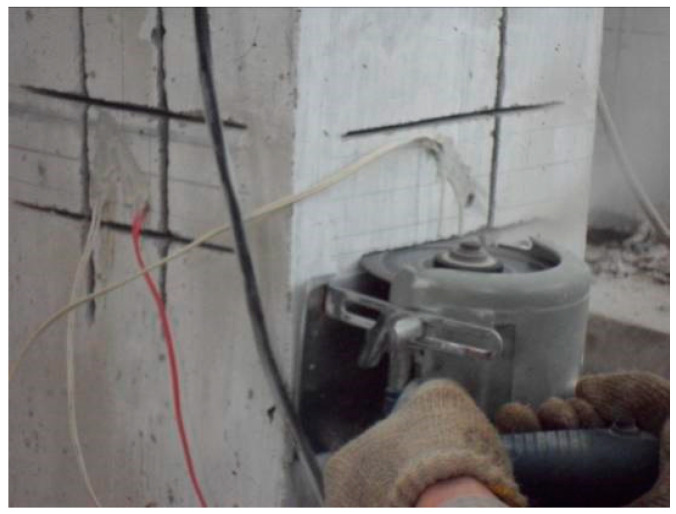
Groove cutting.

Considering several factors, such as concrete immersion in water, temperature fluctuations, and machine cutting disturbance influencing the accuracy of the test results, dry cutting was performed without the use of a water-cooled cutting machine to minimize potential disturbance. Data acquisition was carried out when the test area reached room temperature, and the impact of mechanical cutting on the test area was modified to correct the final test data. Figure 12 shows the process of groove cutting.

### 4.3. Numerical Simulation

**Figure 13:**
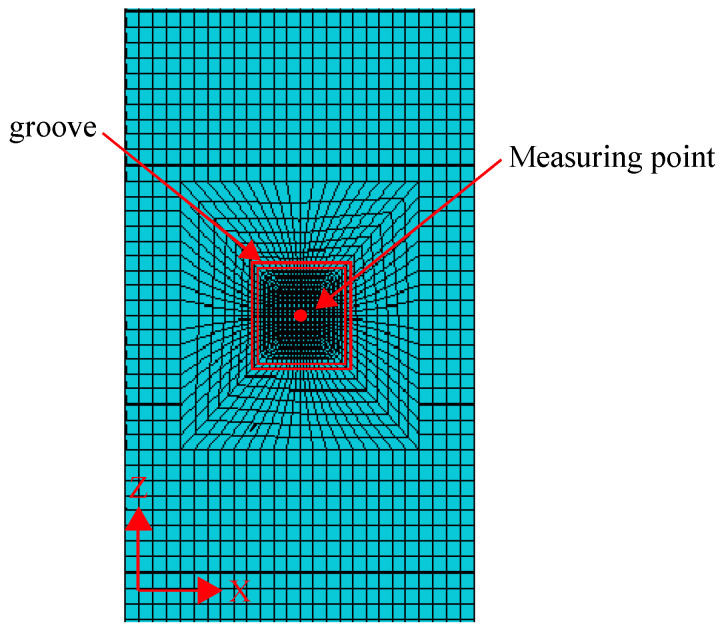
Meshing of the FE model.

A finite element (FE) model of reinforced concrete with dimensions of 250 mm × 250 mm × 700 mm is established using ANSYS 8.1 software to predict the test results. The solid element SOLID45 is applied to model the longitudinal reinforcement, the one-dimensional line element PIPE16 is adopted to simulate the stirrup, and the SOLID65 element is employed to represent the concrete. The Poisson’s ratios for the concrete and the reinforcement are set at 0.2 and 0.3, respectively. The elastic moduli, determined from material property tests, are specified as 2.91 × 10^4^ MPa for the concrete and 2.0 × 10^5^ MPa for the reinforcement. The width of the groove is set at 3 mm, equivalent to the thickness of the cutter blade. In the simulation analysis, the birth and death technique is implemented to simulate the grooving process, that is, the element of the grooving part is killed in each load step to form a slot. The model is divided by hexahedral elements and the encrypted elements are set around the measuring point, as shown in Figure 13.

Before conducting the test, predictions are carried out to analyze various loading conditions and factors influencing the stress release behavior of the reinforced concrete components. The primary focus is on investigating the relationship between the groove depth and the groove side length when the stress in the test area is released to zero, as well as the impact of reinforcement ratio on the released stress values.

In this study, finite element analysis (FEA) is employed as the primary numerical method to investigate these relationships. FEA provides a robust framework for modeling complex structural behaviors, particularly in cases involving irregular geometries and nonlinear material properties. However, it is worth noting that other numerical methods, such as the Finite Difference Method (FDM) and the Bezier Multi-Step Method (BMSM), could also be considered for similar analyses. The FDM, which discretizes partial differential equations into difference equations, is particularly effective for problems with regular geometries and boundary conditions [40]. On the other hand, the BMSM leverages Bezier curves to construct high-order interpolation functions, offering improved accuracy and stability in structural analysis [41]. While FEA is chosen for its versatility and ability to handle the specific complexities of this study, future work could explore the potential of these alternative methods to further validate or complement the findings.

#### 4.3.1. Simulation Results of Release Rule of Normal Stress

**Figure 14:**
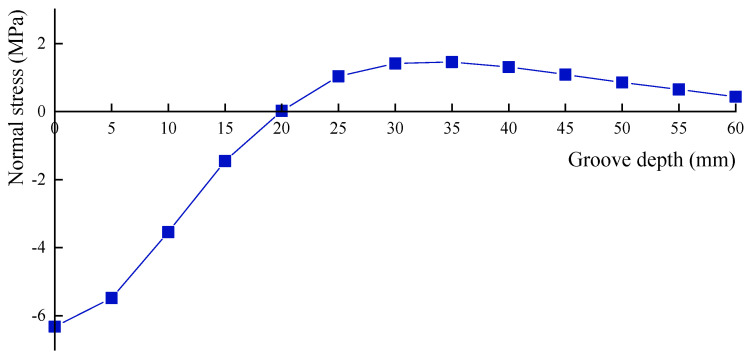
Stress release curve of normal stress.

The relationship between groove depth and groove side length, when normal stress is released to zero, is analyzed. To this end, a uniform axial compressive load of 6.56 MPa is applied to the model, and five different side lengths are selected: 30, 40, 50, 60 and 70 mm. Taking a side length of 60 mm as an example, the normal stresses at the measuring point for different groove depths are calculated and shown in Table 7 and Figure 14.

It is observed that (1) as the groove depth increases, the normal stress at the measuring point is gradually released. The depth is 20 mm when the stress is fully released to zero, which corresponds to one-third of the groove side length. (2) After the stress is fully released to zero, further increasing the groove depth results in the stress initially increasing in the opposite direction to a certain value before decreasing again. This phenomenon occurs because the loading condition changes, transitioning from axial compression to a combination of compression and bending in the grooved region. Consequently, the stress at the measuring point will undergo change from compressive stress to zero stress, and then to tensile stress. As the groove depth continues to increase, the bending effect at the measuring point gradually diminishes, and the tensile stress decreases progressively to zero.

Similarly, the groove depths are determined for other groove side lengths when normal stress is released to zero, as shown in Table 8. It is evident from Table 8 that when the groove depth is one-third of the side length, the normal stress is released to zero.

#### 4.3.2. Simulation Results of Release Rule of Shear Stress

**Figure 15:**
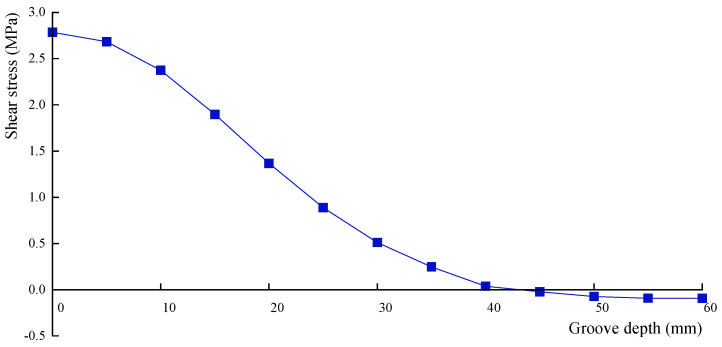
Stress release curve of shear stress.

The relationship between groove depth and groove side length when shear stress is released to zero is analyzed by selecting side length of 30, 40, 50, 60, 70 mm, and a torque of 9.1 kN·m is applied to the model. In the same manner, a side length of 60 mm is taken as an example to calculate the shear stresses at the measuring point for different groove depths, as presented in Table 9 and Figure 15.

It is noted that as the groove depth increases, the shear stress at the measuring point is gradually released. The release rate of shear stress is relatively lower than that of normal stress at the measuring point under the same conditions. The groove depth is 42 mm when the shear stress is fully released to zero by fitting the data in Table 9.

Similarly, the groove depths are determined for other groove side lengths when shear stress is released to zero, as shown in Table 10.

**Figure 16:**
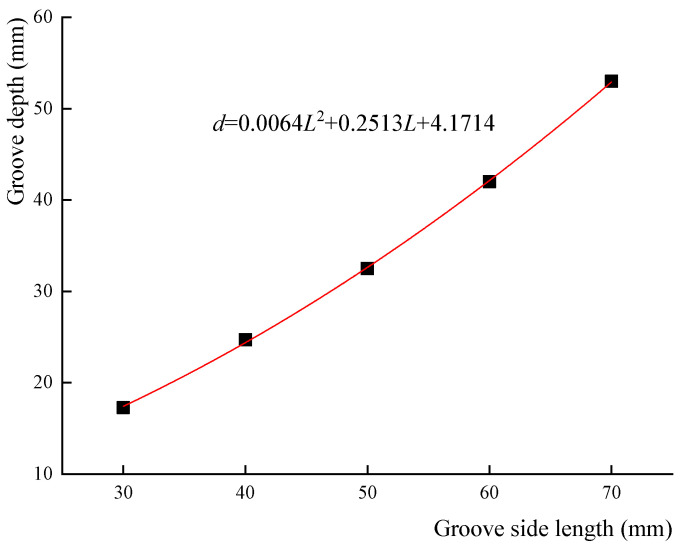
Curve of groove depth versus groove side length when shear stress is released to zero.

Figure 16 illustrates the nonlinear relationship between groove depth and groove side length when shear stress is released to zero. By fitting the data in Table 10, the following equation is obtained:

*d* = 0.0064*L*^2^ + 0.2513*L* + 4.1714 (45)

where *L* is the side length of the square groove and *d* is the groove depth.

#### 4.3.3. Effect of Reinforcement Ratio

The effect of the reinforcement ratio *ρ* on the stress release level is analyzed by selecting the reinforcement ratios of 0% and 2.56%. A groove side length of 60 mm × 60 mm is determined, with a uniform axial compressive load of 9.6 MPa and a torque of 19.8 kN·m applied to the model, respectively, to simulate the stress release behavior of normal stress and shear stress with increasing groove depth under different reinforcement ratios. To normalize the data, the residual strain rate is defined as the ratio of the measured strain value to the initial strain value, and a negative residual strain ratio indicates that the strain increases in the opposite direction after being released to zero. Table 11 presents the relationship between residual strain rate and groove depth under different reinforcement ratios. It is observed that the effect of reinforcement ratio on the stress release level is minimal, indicating the sectional reinforcement ratio could be disregarded in the experimental study.

Based on the above simulation predictions, a standardized square ring groove with dimensions of 60 mm × 60 mm was employed in the test, to investigate the release behavior of normal stress and shear stress, focusing on their relationship with the groove depth, and to validate the feasibility and accuracy of using stress release technology to identify the internal forces of the component’s section.

## 5. Test Results

### 5.1. Disturbance Strain

In the grooving process, three main factors may bring errors to the test results. (1) Concrete immersion in water. When using a ring cutter for grooving, water is typically employed to cool the circular saw, moistening the concrete. This additional moisture can generate additional strain in the concrete. To solve this issue, dry cutting by a concrete cutting machine was applied to prevent concrete immersion. (2) Concrete heating. Mechanical cutting can elevate the temperature of the test area. Following the method recommended in Reference [42], strain data were collected after the concrete temperature in the test area returned to room temperature, typically 2 h after cutting. (3) Concrete disturbance. Grooving with mechanical instruments can induce plastic deformation in the concrete near the groove, resulting in unrecoverable disturbance strain at the measuring point.

Therefore, the test data obtained at the measuring point includes both released and disturbance strains. Given the theoretical challenges in evaluating the magnitude of disturbance strain, an experimental method was employed to accurately measure it. This approach allows for the elimination of test errors arising from disturbance through a correction method.

**Figure 17:**
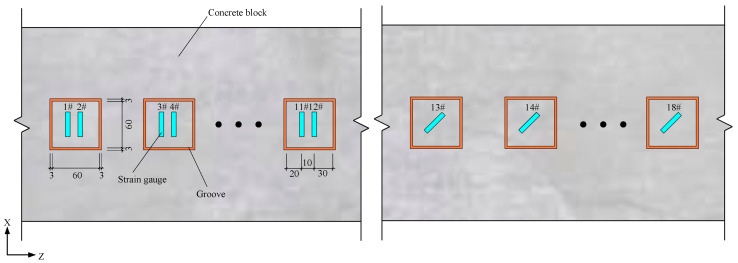
Arrangement of strain gauges for group C1.

In the group C1 test, normal strain and shear strain caused by cutting disturbance were measured by arranging 12 strain measurement zones in the 90° direction (groove depth 20 mm) and six strain measurement zones in the 45° direction (groove depth 42 mm) on each specimen, as presented in Figure 17. After processing the test results, the mean value of the disturbance strain for each specimen was determined, as shown in Table 12. It is evident that as the concrete strength increases, the disturbance strain value decreases for specimens with the same grooving depth.

### 5.2. Release Rule of Normal Stress and Corresponding Internal Force Identification

#### 5.2.1. Release Rule of Normal Stress

**Figure 18:**
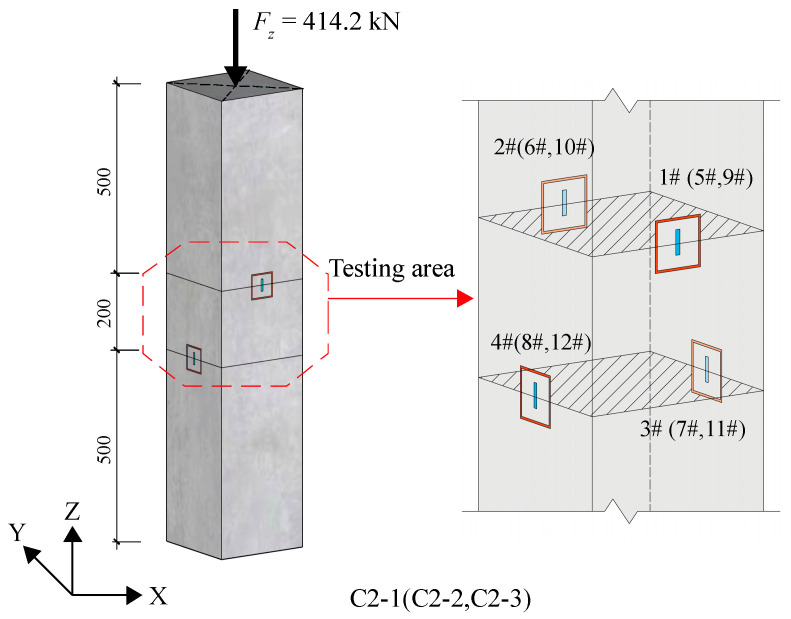
Test diagram of group C2.

In group C2 test, as shown in Figure 18, each specimen had four test areas, resulting in a total of 12 test areas/strain gauges (numbered 1#~12#) across three specimens. Test areas 1#~6# were set up to investigate the release behavior of normal stress. An axial pressure of 414.2 kN was applied to measure the initial compressive strain in the test area. The stress release ring groove was incrementally cut at a depth of 5 mm, and the strain values in the test area were recorded after each cutting until the groove depth reached 40 mm.

**Figure 19:**
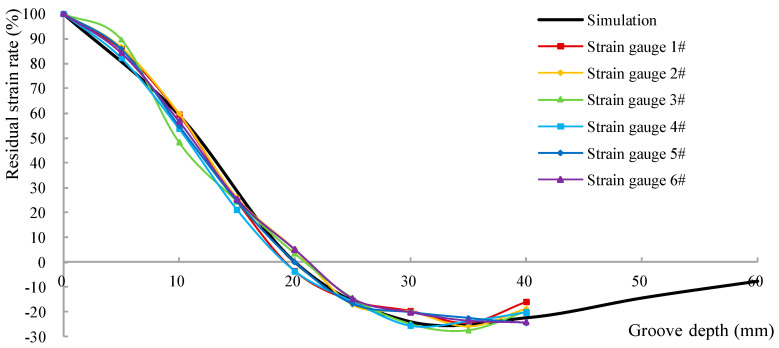
Residual strain rate of normal stress versus groove depth.

To normalize the data, the residual strain rate is defined as the ratio of the measured strain value to the initial strain value. Table 13 and Figure 19 illustrate the relationship between the residual strain rate and the groove depth in the six test areas. The results depicted in Figure 19 demonstrate that the test outcomes align closely with the calculated prediction results, confirming that the released strain corresponds to the original normal strain of the component when the groove depth reaches one-third of the side length of the test area.

#### 5.2.2. Identification of Axial Force and Bending Moment

In the following test on specimens C2-1~3, six additional test areas were established on the other surfaces of the specimens, designed as areas/strain gauges 7#~12#, as presented in Figure 18. The specimens were directly grooved to a depth of 20 mm, and the released strain values at the measured point were recorded. The axial force applied to the specimens was inferred by measured released stress after the modification of the disturbance strain values, as discussed in Section 5.1. The modified data were compared with the loading axial force.

The data collected from strain gauges 7#~12# and the results modified according to Table 12 are shown in Table 14. The calculated axial forces of the specimen sections are presented in Table 15. It is observed that the maximum error in the axial force of one section is 6.90%.

**Figure 20:**
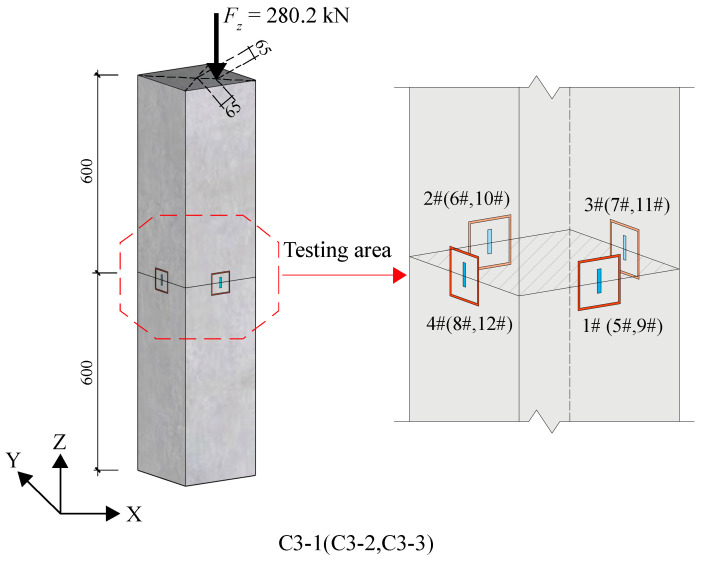
Test diagram of group C3.

Similarly, stress release tests were carried out on specimens C3-1~3, which were subjected to an axial force of 280.2 kN with an eccentricity of 65 mm. This resulted in bidirectional bending moments and axial forces in the identified section, located 600 mm from the vertex of the specimen, as depicted in Figure 20. The internal forces of the section were deduced from the tested strain shown in Table 16 and compared with the loading axial forces and bending moments. The comparison results are summarized in Table 17. It is noted that the error in the internal forces of the sections is within 10%. The maximum error is observed in the axial force of the C3-1 specimen, which is about 8.60%, and the errors in the internal force identification for the other two sections are relatively small.

### 5.3. Release Rule of Shear Stress and Corresponding Internal Force Identification

#### 5.3.1. Release Rule of Shear Stress

**Figure 21:**
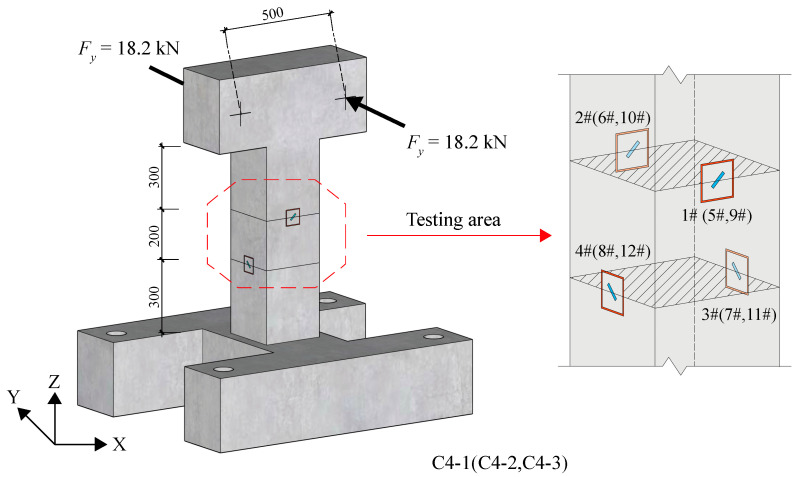
Test diagram of group C4.

**Figure 22:**
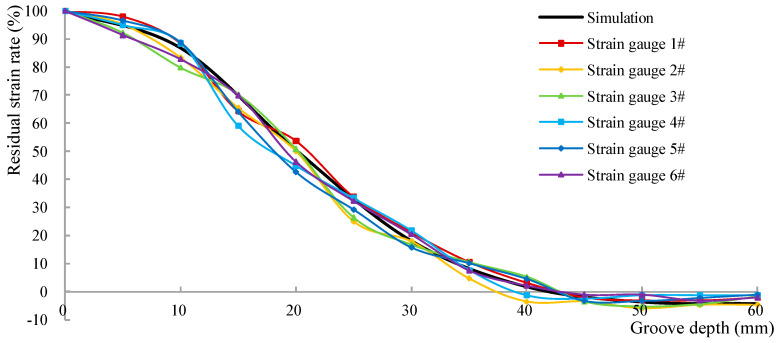
Residual strain rate of shear stress versus groove depth.

In the C4 group, each specimen had four test areas, resulting in a total of 12 test areas/strain gauges (numbered 1#~12#) across the three specimens, as depicted in Figure 21. Test areas 1#~6# were designated to investigate the release behavior of shear stress. A pair of Y-direction forces, each with a magnitude of 18.2 kN, were applied at the loading point, generating a torque of 9.1 kN·m in the cross section to measure the initial compressive strain in the test area. Subsequently, a stress release ring groove was incrementally cut at a depth of 5 mm, and the strain values of the test area after each cutting were recorded until the groove reached a depth of 60 mm. Similar to the methodology outlined in Section 5.2, the residual strain rate is defined as the ratio of the measured strain value to the initial strain value. The relationship between the residual strain rate and the groove depth in the six test areas is shown in Table 18 and Figure 22. It is evident from Figure 22 that the test results are basically consistent with the calculated prediction results, thereby validating the released strain corresponds to the original shear stress of the component when the groove depth reaches 42 mm.

#### 5.3.2. Identification of Torque and Shear Force

In addition to the initial tests on the specimens C4-1~3, six additional test areas were set up on the other surfaces of the specimens, numbered 7#~12#, as shown in Figure 21. These new test areas were directly slotted to a depth of 42 mm, and the released strain value at the measured point was recorded. The torque applied to the specimen was inferred after modifying the disturbance strain values as outlined in Section 5.1. A comparison is made between the identified torque with the loading torque.

Table 19 displays the data collected from strain gauges 7#~12#, with the results modified according to Table 12. The calculated torque for the specimen sections is presented in Table 20. It is noted from the calculation results that the error in the sectional torque ranges from approximately 15% to 20%. Additionally, the accuracy of released shear stress values is observed to be lower than that of normal stress measurements.

**Figure 23:**
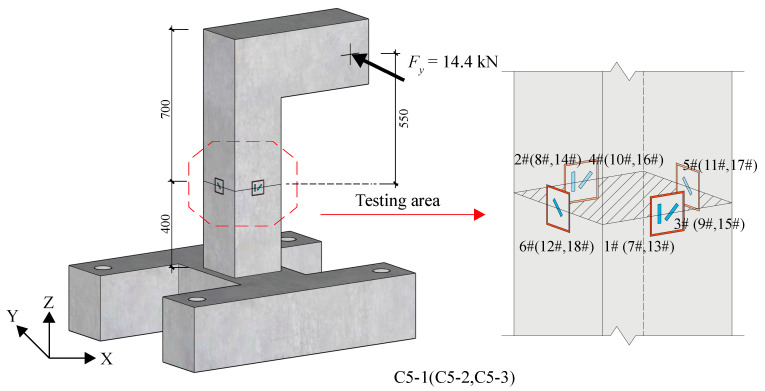
Test diagram of group C5.

Additionally, the stress release test was carried out on specimens C5-1~3, which were subjected to a Y-direction force of 14.4 kN. This results in a bending moment of 7.92 kN·m, a shear force of 14.4 kN and a torque of 7.2 kN·m generated in the identified section, located 550 mm from the loading point, as shown in Figure 23. The test procedure was divided into two steps. First, a groove was directly slotted to a depth of 20 mm to measure the released normal strain data. Subsequently, after the test area returned to room temperature, the groove was further cut to a depth of 42 mm, and the released shear strain data were measured and read. By deducing the internal forces within the specimen section based on the measured strains shown in Table 21, a comparison was made with the actual loading values. The calculation results shown in Table 22 indicate that the identification accuracy of the bending moment within the section is the highest, with identification errors within 10% for two sections. The identification error for the torque within the section is relatively large, with a maximum error reaching 24.3%. Furthermore, the identification error for the shear force within the section is the largest, failing to meet accuracy requirements. This discrepancy is attributed to the low level of released shear strain with a minimum value of −2, which is comparable to the error magnitude. The small value of the identified shear force *F_y_* is due to the fact that the vast majority of its generated shear strain is submerged in the torque-generated shear strain, which cannot be separated from the released data, and therefore its identification is not satisfactory. To address the challenge, future research efforts should focus on error control in stress release technology.

## 6. Conclusions

In this study on stress release technology for identifying internal forces in existing concrete components, several key conclusions can be drawn based on theoretical derivation and experimental investigation:

(1) A detailed derivation of normal stress and shear stress at the midpoint of each side of the section under the action of each internal force component indicates that measuring the normal strain and shear strain values at the midpoint of three sides of a rectangular section enables the identification of the section’s internal forces.
(2) The finite element model used in this study provides a robust description of the relationship between released stress values and groove depth. The groove depth is found to be one-third of the side length of the test area when the normal stress is released to zero, and the groove depth conforms to equations *d* = 0.0064*L*^2^ + 0.2513*L* + 4.1714 when the shear stress is released to zero, which strongly agree with the test results of concrete specimens. This suggests that the finite element model adequately describes the stress distribution and stress release behavior observed in the experiments.
(3) To enhance the accuracy of internal force identification, the influence of concrete immersion in water, heating, and disturbance is considered. Additionally, disturbance strains caused by cutting are measured during the test to obtain modified released strains.
(4) For concrete column specimens subjected to axial compression and bidirectional small eccentricity, the identification errors for axial force and bending moment within the section are less than 10%.
(5) For specimens subjected to pure torsion or combined bending-shear-torsion loads, the identification errors for torque and shear force are a little higher, with the maximum torque identification error reaching 24.3%, and the shear force identification error failing to meet the requirements. The error is attributed to the low level of released shear strain with a minimum value of −2, which is comparable to the error magnitude. Therefore, future research should focus on error control in stress release technology to improve accuracy under these loading conditions.

This study primarily focuses on rectangle grooves in reinforced concrete. Future research will extend experimental and numerical studies to different groove shapes and other concrete types. Additional tests on disk-shaped and cracked disk specimens will be conducted to validate the findings under more complex scenarios.

## Figures and Tables

**Table 1:** Factors *β*_1_ and *υ*_1_ of rectangular section subjected to torsion.

a/b	1	1.2	1.5	1.75	2.0	2.5	3	4	6	8	10	∞
*β*_1_	0.208	0.219	0.231	0.239	0.246	0.258	0.267	0.282	0.299	0.307	0.312	0.333
*υ*_1_	1.000	0.930	0.859	0.820	0.795	0.766	0.753	0.745	0.743	0.742	0.742	0.742

**Table 2:** Shear stress factor λ of the rectangular section.

a/b	0.6	0.8	1.0	1.2	1.4	1.6	1.8	2.0
λ	0.125	0.067	0.035	0.019	0.010	0.005	0.003	0.002

**Table 3:** Torsional position factor *η_i_* of steel bars.

*b* (mm)	Steel Bars			
	1#	2#	3#	4#
100	0.002	0.130	0.195	0.200
200	0.002	0.264	0.523	0.593
250	0.002	0.269	0.566	0.665
300	0.002	0.270	0.589	0.709
350	0.002	0.269	0.602	0.738
400	0.002	0.269	0.611	0.757
450	0.002	0.268	0.617	0.770
500	0.002	0.268	0.621	0.779

**Table 4:** Shear position factor *λ_i_* of steel bars.

*b* (mm)	Steel Bars			
	1#	2#	3#	4#
100	0.291	0.291	0.745	0.994
200	0.283	0.289	0.738	0.983
250	0.278	0.289	0.735	0.978
300	0.273	0.289	0.732	0.973
350	0.267	0.288	0.730	0.969
400	0.261	0.288	0.727	0.964
450	0.255	0.288	0.725	0.960
500	0.249	0.287	0.723	0.956

**Table 5:** Test contents.

Specimen Group	Force State	Concrete Strength Grade	Design Load	Test Objective
C1	No external force	C20(C1-1) C30(C1-2) C40(C1-3)	—	obtain the disturbance strain during machine cutting; relationship between the disturbance strain and the concrete strength.
C2	Axial compression	C30(C2-1~3)	*F_z_* = −437.5 kN	release rule of normal stress at the measuring point with the groove depth; axial force identification.
C3	Bidirectional small eccentric compression	C30(C3-1~3)	*F_z_* = −240 kN *M_x_* = 15.6 kN·m *M_y_* = 15.6 kN·m	axial force and bending moment identification.
C4	Pure torsion	C30(C4-1~3)	*M_z_* = 6.5 kN·m	release rule of shear stress at the measuring point with the groove depth; torque identification.
C5	Bending-shear-torsion	C30(C5-1~3)	*M_x_* = −4.95 kN·m *F_y_* = 9 kN *M_z_* = 4.5 kN·m	bending moment, shear and torque identification.

Note: There is a certain difference between the applied load and the design load in the test. The applied load is recorded to analyze errors below.

**Table 6:** Measured material properties.

Material	Compressive Strength (MPa)	Young’s Modulus (MPa)	Shear Modulus (MPa)	Poisson’s Ratio
C30 Concrete	32.1	2.91 × 10^4^	1.21 × 10^4^	0.2
HRB335 Steel	—	2.00 × 105	7.69 × 10^4^	—

**Table 7:** Normal stress at different groove depths.

Groove Depth (mm)	0	5	10	15	20	25	30	35	40	45	50	55	60
Normal stress (MPa)	−6.326	−5.483	−3.545	−1.458	0.019	1.037	1.418	1.455	1.309	1.088	0.854	0.648	0.438

**Table 8:** Groove depths for different groove side lengths when normal stress is released to zero.

Groove Side Length (mm)	30	40	50	60	70
Groove depth (mm)	10	13.3	16.7	20.0	23.3
Depth/side length	0.33	0.33	0.33	0.33	0.33

**Table 9:** Shear stress at different groove depths.

Groove Depth (mm)	0	5	10	15	20	25	30	35	40	45	50	55	60
Shear stress (MPa)	2.785	2.683	2.374	1.897	1.367	0.887	0.511	0.247	0.037	−0.023	−0.073	−0.092	−0.093

**Table 10:** Groove depths for different groove side lengths when shear stress is released to zero.

Groove Side Length (mm)	30	40	50	60	70
Groove depth (mm)	17.3	24.7	32.5	42.0	53.0
Depth/side length	0.58	0.62	0.65	0.70	0.76

**Table 11:** Relationship between residual strain rate (%) and groove depth under different reinforcement ratios.

	Groove Depth (mm)	0	10	20	30	40	50	60
Reinforcement Ratio								
Normal strain	*ρ* = 2.56%	100.0	59.1	0.0	−23.7	−22.3	−14.3	−7.5
	*ρ* = 0.00%	100.0	59.2	0.0	−23.9	−22.5	−14.5	−7.6
Shear strain	*ρ* = 2.56%	100.0	86.6	50.0	17.7	1.5	−3.8	−4.2
	*ρ* = 0.00%	100.0	86.8	50.5	18.1	1.7	−3.7	−4.2

**Table 12:** Mean values of disturbance strain (*με*).

Specimen/Concrete Strength Grade	C1-1/C20		C1-2/C30		C1-3/C40	
Groove depth	20 mm	42 mm	20 mm	42 mm	20 mm	42 mm
Normal strain	−24	—	−17	—	−13	—
Shear strain	—	−43	—	−34	—	−23

**Table 13:** Residual strain rate of normal stress under different groove depths (%).

	Groove Depth (mm)	0	5	10	15	20	25	30	35	40
Number										
1#		100	85.7	59.5	24.4	−3.57	−15.5	−19.6	−24.4	−16.1
2#		100	86.5	59.8	25.9	5.18	−17.1	−19.9	−25.9	−18.7
3#		100	89.6	48.3	24.4	3.48	−14.4	−24.9	−27.4	−19.9
4#		100	82.1	53.8	21.1	−3.59	−15.7	−25.6	−23.8	−20.2
5#		100	85.8	54.9	24.5	0.00	−16.7	−20.1	−22.5	−24.5
6#		100	84.3	56.7	25.3	5.06	−14.6	−20.2	−23.6	−24.2

**Table 14:** Results of calculated and modified released strain values (*με*) of group C2.

Strain Gauge Number	Released Strain	Disturbance Strain Correction	Modified Released Strain
7#	−204	−17	−221
8#	−149	−17	−166
9#	−183	−17	−200
10#	−204	−17	−221
11#	−208	−17	−225
12#	−158	−17	−175

**Table 15:** Results of the axial force identification of group C2.

Specimen Number	Actual Axial Force	Identified Axial Force	Error
C2-1	*F_z_* = 414.2 kN	*F_z_* = 385.6 kN	6.90%
C2-2	*F_z_* = 414.2 kN	*F_z_* = 419.4 kN	1.30%
C2-3	*F_z_* = 414.2 kN	*F_z_* = 398.5 kN	3.80%

**Table 16:** Results of calculated and modified released strain values (*με*) of group C3.

Strain Gauge Number	Released Strain	Disturbance Strain Correction	Modified Released Strain
1#	−315	−17	−332
2#	92	−17	75
3#	−324	−17	−341
4#	68	−17	51
5#	−315	−17	−332
6#	69	−17	52
7#	−339	−17	−356
8#	85	−17	68
9#	−304	−17	−321
10#	78	−17	61
11#	−286	−17	−303
12#	82	−17	65

**Table 17:** Results of the internal force identification of group C3.

Specimen Number	Actual Internal Force	Identified Internal Force	Error
C3-1	*F_z_* = 280.2 kN	*F_z_* = 256.1 kN	8.60%
	*M_x_* = 18.2 kN·m	*M_x_* = 16.9 kN·m	7.10%
	*M_y_* = 18.2 kN·m	*M_y_* = 17.5 kN·m	3.80%
C3-2	*F_z_* = 275.5 kN	*F_z_* = 287.0 kN	4.20%
	*M_x_* = 17.9 kN·m	*M_x_* = 18.2 kN·m	1.80%
	*M_y_* = 17.9 kN·m	*M_y_* = 16.5 kN·m	7.80%
C3-3	*F_z_* = 257.7 kN	*F_z_* = 237.1 kN	8.00%
	*M_x_* = 16.8 kN·m	*M_x_* = 15.8 kN·m	6.00%
	*M_y_* = 16.8 kN·m	*M_y_* = 16.4 kN·m	2.40%

**Table 18:** Residual strain rate of shear stress under different groove depths (%).

	Groove Depth (mm)	0	5	10	15	20	25	30	35	40	45	50	55	60
Number														
1#		100	97.9	88.4	64.2	53.7	33.7	21.1	10.5	3.16	−2.11	−3.16	−3.16	−2.11
2#		100	95.2	83.3	65.5	50.0	25.0	17.9	4.76	−3.57	−3.57	−5.95	−4.76	−4.76
3#		100	92.1	79.8	70.2	50.9	26.3	16.7	10.5	5.26	−3.51	−5.26	−4.39	−1.75
4#		100	94.9	88.5	59.0	44.9	33.3	21.8	7.69	−1.28	−2.56	−1.28	−1.28	−1.28
5#		100	96.6	88.8	64.0	42.7	29.2	15.7	10.1	4.49	−3.37	−3.37	−2.25	−1.12
6#		100	91.4	82.8	69.9	46.2	32.3	20.4	7.53	2.15	−1.08	−1.08	−3.23	−2.15

**Table 19:** Results of calculated and modified released strain values (*με*) of group C4.

Strain Gauge Number	Released Strain	Disturbance Strain Correction	Modified Released Strain
7#	101	−34	67
8#	−72	−34	−106
9#	119	−34	85
10#	−80	−34	−114
11#	103	−34	69
12#	−80	−34	−114

**Table 20:** Results of the torque identification of group C4.

Specimen Number	Actual Torque	Identified Torque	Error
C4-1	*M_z_* = 9.1 kN·m	*M_z_* = 7.3 kN·m	19.80%
C4-2	*M_z_* = 9.1 kN·m	*M_z_* = 8.4 kN·m	7.70%
C4-3	*M_z_* = 9.1 kN·m	*M_z_* = 7.7 kN·m	15.40%

**Table 21:** Results of calculated and modified released strain values (*με*) of group C5.

Strain Gauge Number	Released Strain	Disturbance Strain Correction	Modified Released Strain
1#	95	−17	78
2#	−76	−17	−93
3#	76	−34	42
4#	−12	−34	−46
5#	79	−34	45
6#	−10	−34	−44
7#	86	−17	69
8#	−88	−17	−105
9#	129	−34	95
10#	−13	−34	−47
11#	82	−34	48
12#	−5	−34	−39
13#	89	−17	72
14#	−74	−17	−91
15#	68	−34	34
16#	−2	−34	−36
17#	77	−34	43
18#	−2	−34	−36

**Table 22:** Results of the internal force identification of group C5.

Specimen Number	Actual Internal Force	Identified Internal Force	Error
C5-1	*M_x_* = 7.92 kN·m	*M_x_* = 7.36 kN·m	7.10%
	*M_z_* = 7.2 kN·m	*M_z_* = 6.61 kN·m	8.20%
	*F_y_* = 14.4 kN	*F_y_* = 0.49 kN	—
C5-2	*M_x_* = 7.92 kN·m	*M_x_* = 7.49 kN·m	5.40%
	*M_z_* = 7.2 kN·m	*M_z_* = 8.95 kN·m	24.30%
	*F_y_* = 14.4 kN	*F_y_* = 4.43 kN	—
C5-3	*M_x_* = 7.92 kN·m	*M_x_* = 7.01 kN·m	11.50%
	*M_z_* = 7.2 kN·m	*M_z_* = 5.72 kN·m	20.60%
	*F_y_* = 14.4 kN	*F_y_* = 3.45 kN	—

## Data Availability

The original contributions presented in this study are included in the article. Further inquiries can be directed to the corresponding author.
